# KLLN-mediated DNA damage-induced apoptosis is associated with regulation of p53 phosphorylation and acetylation in breast cancer cells

**DOI:** 10.1038/s41420-018-0094-x

**Published:** 2018-09-11

**Authors:** Madhav Sankunny, Charis Eng

**Affiliations:** 1 Genomic Medicine Institute, Cleveland Clinic, Cleveland, OH 44195 USA; 2 Lerner Research Institute, Cleveland Clinic, Cleveland, OH 44195 USA; 3 Taussig Cancer Institute, Cleveland Clinic, Cleveland, OH 44195 USA; 4 Department of Genetics and Genome Sciences, Case Western Reserve University, Cleveland, OH 44106 USA; 5 Germline High Risk Focus Group, CASE Comprehensive Cancer Center, Case Western Reserve University, Cleveland, OH 44106 USA

**Keywords:** Breast cancer, Breast cancer, DNA damage response

## Abstract

KLLN is a target of p53 involved in S-phase cell cycle regulation deemed necessary and sufficient for p53-mediated apoptosis. Germline promoter hypermethylation of *KLLN* is associated with a cancer-predisposition syndrome, Cowden syndrome. KLLN’s DNA-binding ability is associated with transcription regulation and maintenance of genomic stability. Here, we report on KLLN’s role in DNA damage response (DDR) mediated through apoptosis in breast cells with and without a cancer phenotype. *KLLN* expression was upregulated after doxorubicin-induced DNA damage and this upregulation can be abrogated using RNAi-mediated gene silencing. Silencing *KLLN* after doxorubicin treatment effected DDR shown by decreased γ-H2AX foci and expression, and apoptosis assessed by decreased frequency of apoptotic nuclei and decreased expression of definitive markers of apoptosis. Contrary to expectations, there was no change in cell cycle regulation after *KLLN* silencing. These results were observed in breast cells with wildtype and mutant p53. At early timepoints after doxorubicin treatment, knocking down *KLLN* resulted in decreased Ser15-phosphorylation of p53 but not Thr68-phosphorylation of CHK2 or the phosphorylation of upstream regulators such as ATM and ATR. Interestingly, a second pathway for p53 activation was also affected by knockdown of *KLLN*. After doxorubicin treatment, Thr454-phosphorylation of DBC1, required to inhibit deacetylation of p53 by SIRT1, was decreased and therefore acetylation of p53 was also decreased with *KLLN* knockdown. Therefore, our observations suggest that KLLN’s role in DNA damage-induced apoptosis is likely independent of p53 and is associated with a two-pronged regulation of p53 activation.

## Introduction

An immediate and effective response to DNA damage induced by both exogenous and endogenous factors is a critical cellular function required for the maintenance of homeostasis. Perturbations in the DNA damage response (DDR) resulting in decreased apoptosis or cell senescence and genomic instability can lead to carcinogenesis and cancer progression. Tumor suppressor genes are key regulators of DDR, and therefore, frequently mutated in cancers. Germline mutations of *PTEN*, a tumor suppressor gene, is responsible for ~25% of an autosomal dominant cancer-predisposition syndrome, Cowden syndrome characterized by increased risks of breast, thyroid, endometrial, and kidney malignancies^1,2^. Germline hypermethylation in the promoter of another putative tumor suppressor gene, *KLLN*, which shares a bidirectional promoter with *PTEN*, contributes to CS in up to 35% of *PTEN* mutation negative cases and is associated with increased prevalence of breast and renal cell carcinomas^3–5^. Somatic *KLLN* deletions were found in 21% of breast carcinomas in The Cancer Genome Atlas (TCGA), and decreased KLLN expression is associated with increased tumor grade in breast carcinomas vs. adjacent normal tissue^5,6^. This suggests lack of KLLN could be involved in both cancer susceptibility and sporadic carcinogenesis.

KLLN was first reported in 2008 while researchers were looking for targets of p53 involved in S-phase checkpoint control. KLLN was described as a tumor suppressor protein that is both necessary and sufficient for p53-mediated apoptosis in colon cancer cell lines^7^. KLLN is localized to 10q23 and shares a transcription start site with *PTEN*^3,7^. Transcription of both these genes are regulated by p53 and both have known p53-binding sites on their promoters^7,8^. KLLN function is associated with the regulation of cell growth with KLLN overexpression resulting in increased cell death, whereas KLLN knockdown results in increased cellular proliferation, colony formation, and migration in breast and prostate cancer cell lines^6,7,9^. KLLN is also a high affinity DNA-binding protein and is known to bind the promoter of genes such as *TP53* and *CHK1*, and regulate their expression^5,7,9^. Preliminary integrative analyses from a genome-wide ChIP-seq and RNA-seq revealed a global binding pattern and transcriptional regulatory function for KLLN^10^. KLLN expression positively correlates with H3K9 methyltransferase activity and H3K9 trimethylation, and therefore, was required for the maintenance of pericentric heterochromatin and genomic stability^10^. This suggests that KLLN could have a role in DDR.

Initially, KLLN was thought to be an effector molecule downstream of p53 activation that has a role in S or G2-M phase cell cycle checkpoint control in response to genotoxic stress and stalled replication forks^5,7^. Variations in *KLLN* have been reported to lead to G2 checkpoint dysfunction^5^. That *KLLN* as a downstream effector of p53 was called into question when KLLN was found to act as a transcription factor which regulates p53 and CHK1 expression^5,6,9^. To date, there has been no concrete evidence supporting the hypothesis that loss/lack of KLLN expression can result in dysfunction of cell cycle regulation especially at the S or G2-M checkpoints. Therefore, in this context, we demonstrate here that KLLN has a role in DDR especially in DNA damage-induced apoptosis that may be independent of p53-mediated regulation of KLLN function. We also show that contrary to current belief, KLLN is able to regulate p53 activation through the regulation of two parallel pathways.

## Results

### Increased KLLN expression after doxorubicin-induced DNA damage is abrogated by RNAi-mediated silencing of KLLN expression

**Fig. 1:**
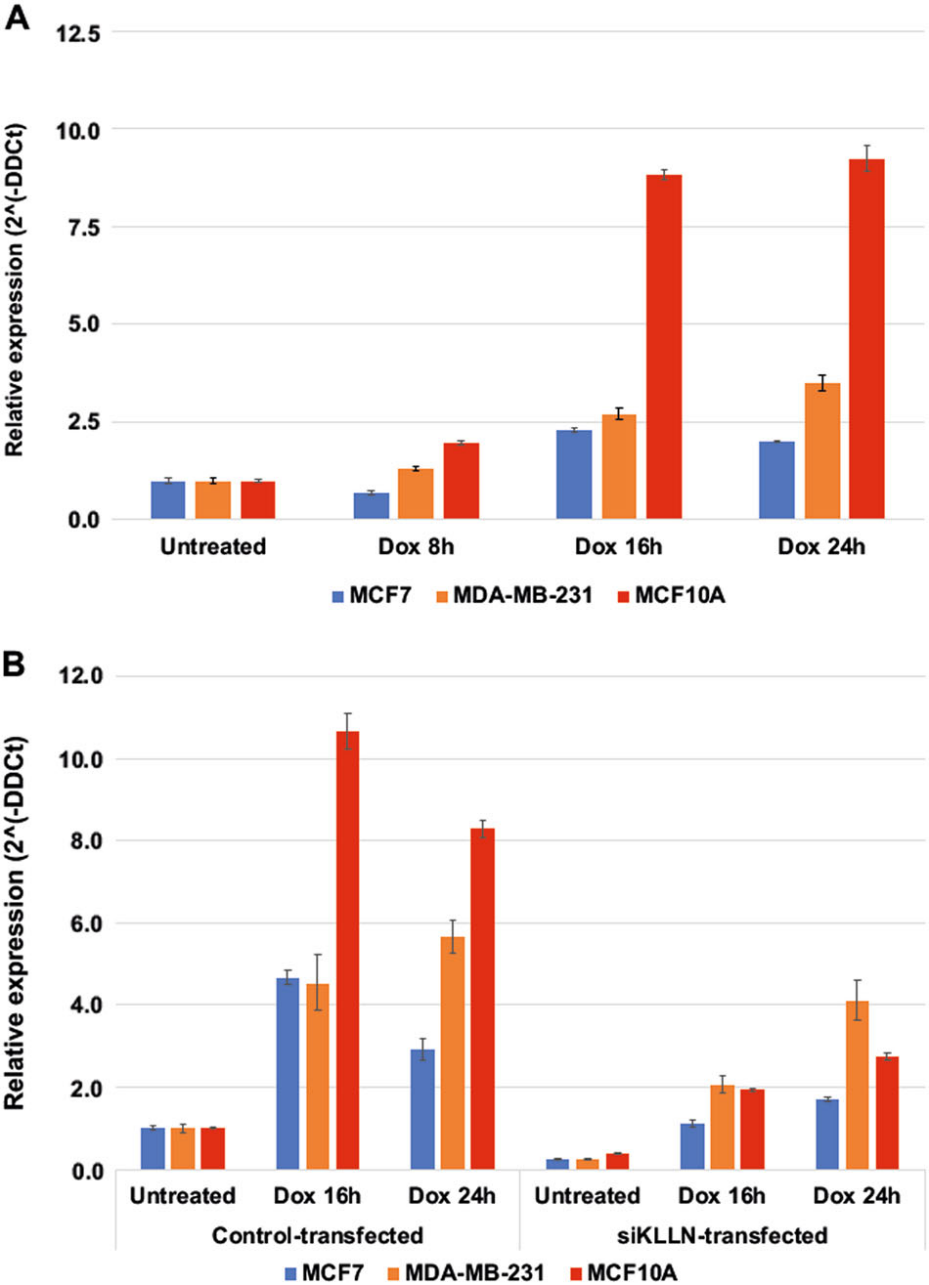
Expression of *KLLN* in response to DNA damage-induced by doxorubicin with and without RNAi-mediated gene silencing. **a** Graph of relative KLLN expression at different timepoints after doxorubicin treatment shows marked increase in *KLLN* expression at 16 h and 24 h after treatment. Values represent mean ± SD. **b** Graph of relative KLLN expression after KLLN knockdown and doxorubicin treatment. The increase observed with doxorubicin treatment alone was decreased after RNAi-mediated KLLN knockdown. Values represent mean ± SD

To induce DNA damage, we treated our cells with doxorubicin hydrochloride. Doxorubicin is an anthracycline antitumor antibiotic that intercalates with DNA and stops replication^11^. It is also known to inhibit the progression of the topoisomerase II enzyme and induces histone eviction at the chromatin^12,13^. Doxorubicin can create both double-strand breaks (DSB) as well as single stand breaks (SSB)^14,15^. On the basis of pilot experiments, a 2 µM dose of doxorubicin was deemed to cause an appropriate amount of damage for our studies. We assessed *KLLN* gene expression at three timepoints (8, 16 and 24 h) after treatment with doxorubicin in all three breast cell lines and observed an increase in *KLLN* expression at 16 and 24 h (Fig. 1a). MCF10A cells showed the largest fold change (>8) at both timepoints tested. We used siRNA to silence *KLLN* expression and assessed the effect of this treatment on *KLLN* expression after treatment with doxorubicin. We observed that the increase in *KLLN* expression observed after doxorubicin treatment was abrogated after siRNA treatment. In MCF7 and MCF10A cells, there was more than a 50% decrease in expression at both timepoints tested (Fig. 1b). This provided us a viable model to test DDR to doxorubicin-induced damage in the absence of KLLN expression.

### RNAi-mediated silencing of KLLN after doxorubicin treatment leads to decreased response to DNA damage and increased genomic instability

**Fig. 2:**
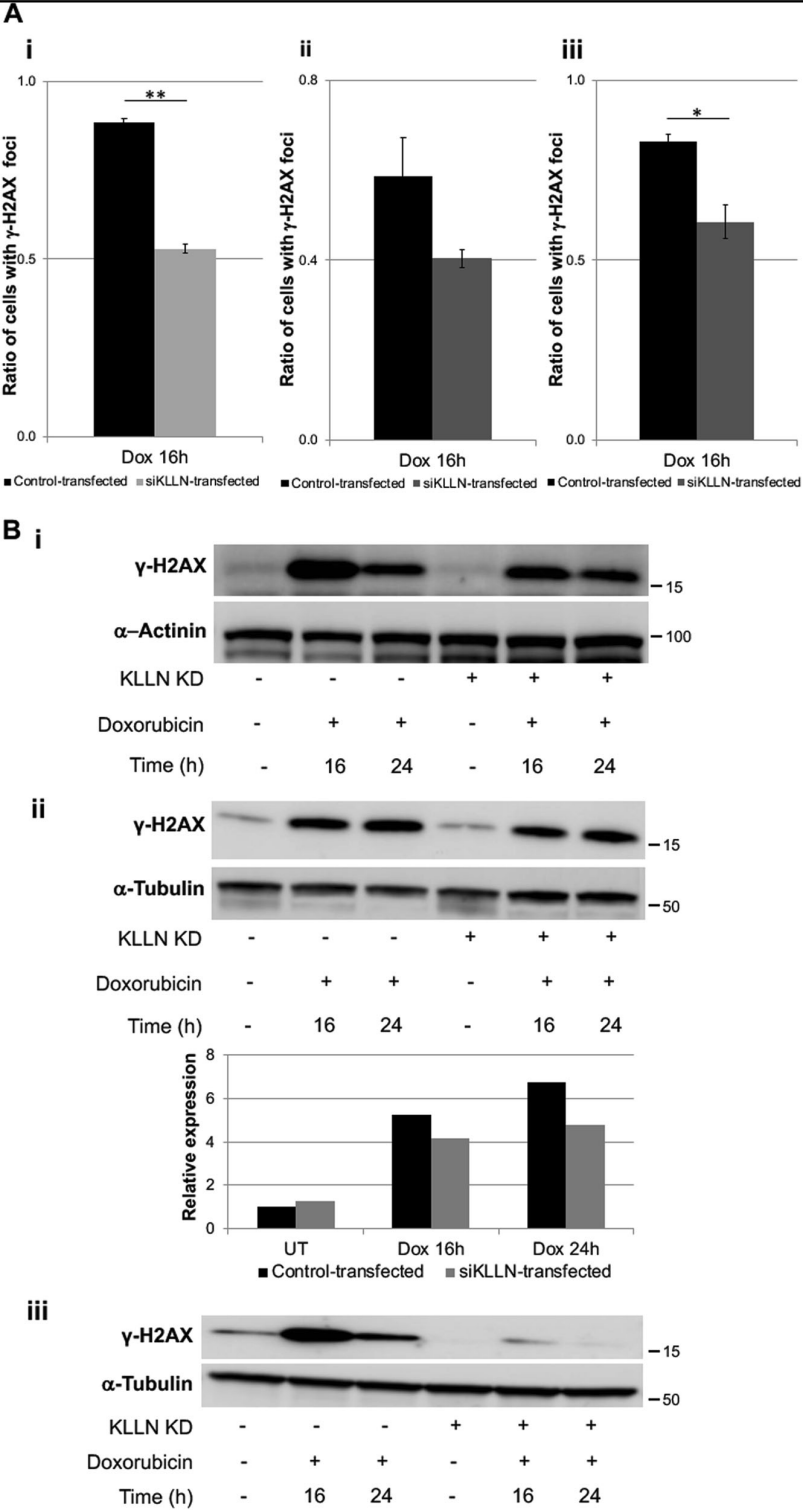
Knockdown of *KLLN* expression results in diminished response to DNA damage in breast cells. **a** RNAi-based knockdown of *KLLN* results in decreased frequency of γ-H2AX foci after doxorubicin-induced DNA damage in MCF7 (i), MCF10A (ii), and MDA-MB-231 (iii) cells. Values represent mean ± SD. **p value < 0.005, *p value < 0.05. **b** γ-H2AX expression in response to DNA damage-induced by doxorubicin was also decreased after knockdown of *KLLN* expression in MCF7 (i), MCF10A (ii), and MDA-MB-231 (iii) cells

An initial response to DNA damage resulting in DSB or SSB is the phosphorylation of the histone variant H2AX at Serine residue 139^16,17^. This phosphorylation is part of a positive feedback loop resulting in increased H2AX phosphorylation. These phosphorylated H2AX or γ-H2AX molecules aggregate at sites of DNA damage forming foci that are responsible for the recruitment of downstream transducer and effector molecules^18–20^. We assessed the effect of siRNA-based *KLLN* silencing on the response to DNA damage using γ-H2AX foci and expression as a marker. Knocking down *KLLN* expression resulted in decreased DDR as reflected by decreased γ-H2AX foci at 16 h after treatment in all three breast cell lines tested (Fig 2a). γ-H2AX expression assessed by Western blotting was also decreased at 16 h and 24 h after treatment in all cell lines tested (Fig. 2b). A consequence of an inefficient response after DNA damage is the propagation of cells with genomic instability. As such, we assessed micronuclei frequency as a marker of genomic instability. At 16 and 24 h after treatment with doxorubicin in MDA-MB-231 cells, we observed that knockdown of KLLN expression resulted in increased micronuclei frequency (Fig S1). These results are suggestive of an incomplete or inefficient DDR in the absence of KLLN expression.

### RNAi-mediated silencing of KLLN after doxorubicin-induced DNA damage inhibits the apoptotic response but not cell cycle regulation

The effective clearing of DNA damage can be achieved either by cell cycle regulation resulting in arrest of the cell cycle followed by DNA repair, or in the case of irreversible damage, by the induction of apoptosis^21^. We used propidium iodide staining followed by flow cytometry to assess cell cycle regulation. For the cell cycle regulation experiments, we used lower doses (500 or 1000 nM) of doxorubicin so as not to induce irreversible damage and apoptosis. We show that there was no difference in accumulation of cells in the different phases of the cell cycle after doxorubicin treatment between the un-transfected, control-transfected or the siKLLN-transfected groups in any of the cell lines tested (Fig S2). Contrary to prior literature suggesting a role for KLLN in S-phase cell cycle regulation^7^, increased KLLN expression following doxorubicin-induced DNA-damage did not result in S-phase cell cycle arrest. Therefore, the role of KLLN in DDR is likely not mediated through regulation of the cell cycle.

**Fig. 3:**
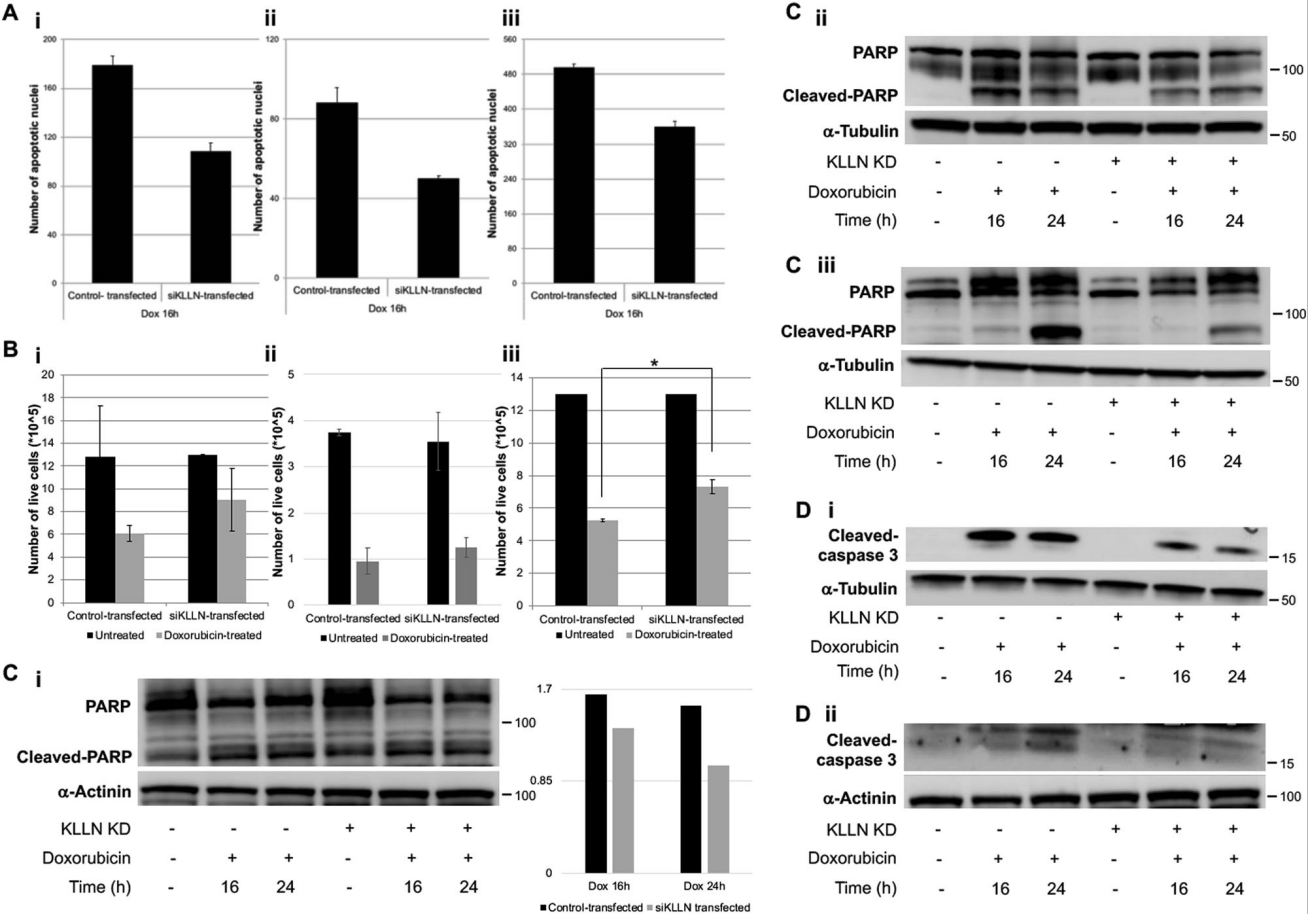
KLLN has a role in apoptosis regulation but not cell cycle regulation after DNA damage. **a** TUNEL assay measuring frequency of apoptotic nuclei showed that after doxorubicin-induced DNA damage, there are fewer apoptotic nuclei in breast cells [(i) MCF7, (ii) MCF10A and (iii) MDA-MB-231] with knockdown of *KLLN* expression. Values represent mean ± SD. **b** Cell viability assays showed increased proliferation of breast cells [(i) MCF7, (ii) MCF10A and (iii) MDA-MB-231] with knockdown of *KLLN* expression after DNA damage. Values represent mean ± SD. *p value < 0.05. **c** Immunoblotting for cleaved PARP, a marker of apoptosis showed decrease in cleavage after doxorubicin-induced DNA damage in breast cells [(i) MCF7, (ii) MCF10A and (iii) MDA-MB-231] with knockdown of *KLLN* expression. **d** Immunoblotting for cleaved caspase-3 after doxorubicin-induced DNA damage in breast cells MCF10A (i) and MDA-MB-231 (ii) showed decrease in cleavage with knockdown of *KLLN* expression

Next, we tested KLLN’s role in apoptosis using TUNEL assay to assess frequency of apoptotic nuclei and using cell viability assay to assess proliferative capability of the cells after doxorubicin-induced DNA damage. TUNEL assay revealed that knocking down *KLLN* expression in all three lines at 16 h after doxorubicin treatment resulted in decreased frequency of apoptotic nuclei (Fig. 3a). We also showed that there was a parallel increase in cell viability with decreased apoptosis when *KLLN* expression was knocked down. Increased cell viability was observed at 16 h after treatment in all lines with knockdown of *KLLN* (Fig. 3b). Therefore, these observations suggest that KLLN may have a role in apoptosis regulation, but not cell cycle regulation, in response to DNA damage. To confirm the role of KLLN in apoptosis induction, we assessed the expression of such markers of apoptosis as cleavage of caspase-3 and PARP using immunoblotting. We observed that in all three lines tested, lack of *KLLN* expression after treatment with doxorubicin resulted in decreased cleavage of PARP (Fig. 3c). Decrease in cleavage of caspase-3 with lack of *KLLN* expression was observed only in MDA-MB-231 and MCF10A lines (Fig. 3d), since MCF7 cell line is known to be deficient for caspase-3^22,23^. These data suggest that KLLN has an important role in apoptosis regulation in response to DNA damage.

### Loss of KLLN expression decreases phosphorylation of p53 at early timepoints after doxorubicin-induced DNA damage

**Fig. 4:**
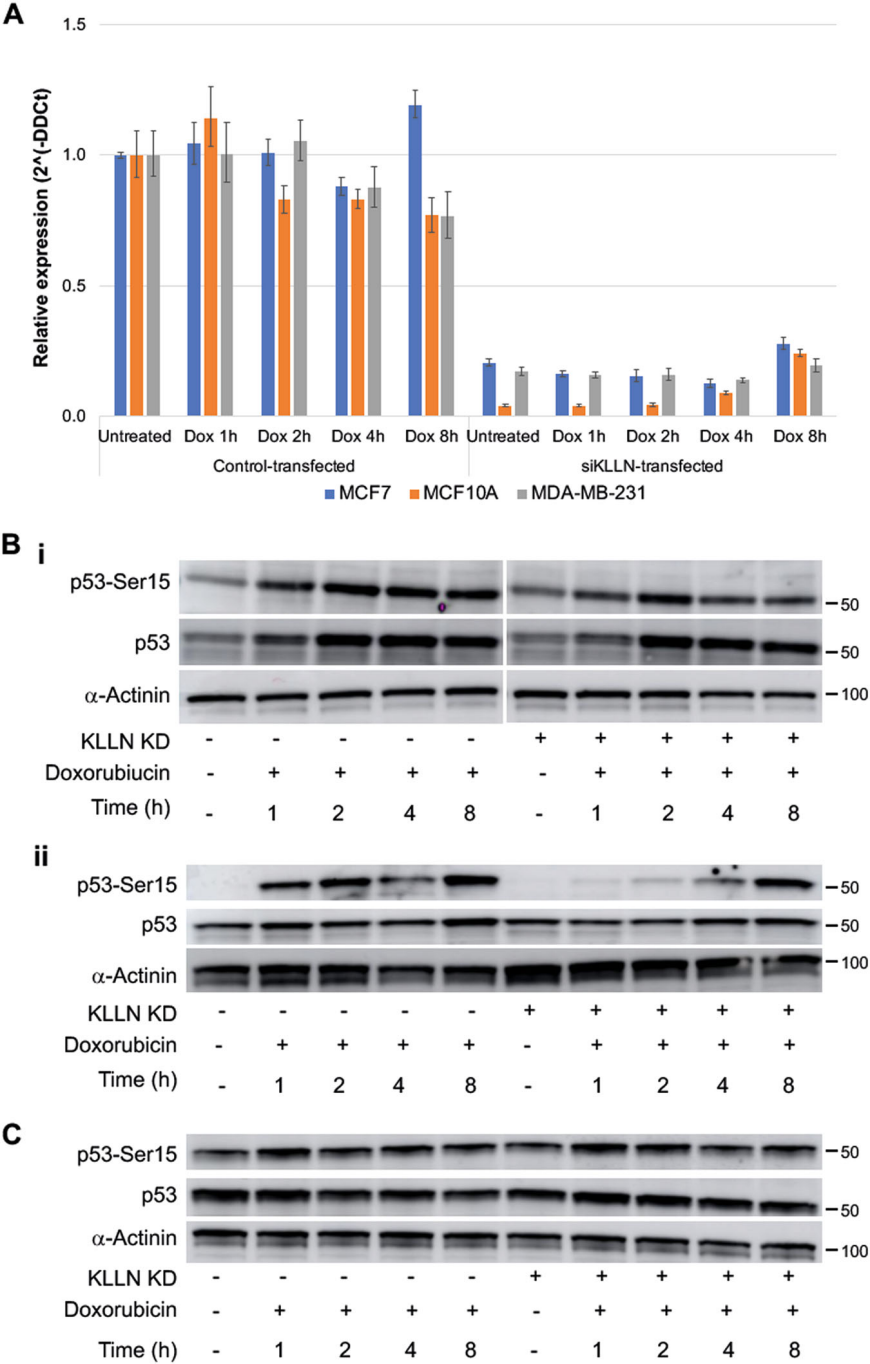
KLLN effects phosphorylation of p53 but not phosphorylation of CHK2 after DNA damage in breast cells. **a** RNAi-mediated silencing of *KLLN* was able to maintain decreased *KLLN* expression through early timepoints (1, 2, 4, and 8 h) after doxorubicin-induced DNA damage in all three breast cell lines. Values represent mean ± SD. **b** Immunoblotting for Ser15-phosphorylation of p53 showed that knockdown of *KLLN* expression resulted in decreased p53 phosphorylation in MCF7 (i) and MCF10A (ii) cells. Total p53 expression was assessed and found to be unaffected due to knockdown of *KLLN* expression. **c** Immunoblotting for Ser15-phosphorylation of p53 in MDA-MB-231 cells after doxorubicin-induced DNA damage showed no effect on this phosphorylation with knockdown of *KLLN* expression

Since response to DNA damage is immediate, we evaluated the effect of silencing *KLLN* expression on the early induction of DDR. First, using qRT-PCR we confirmed that knocking down *KLLN* expression was effective at early timepoints. RNAi-mediated silencing was able to maintain the knockdown (>80%) of *KLLN* expression in all three lines at early timepoints (1 and 2 h) and later timepoints (4 and 8 h) after doxorubicin treatment (Fig. 4a). In MCF10A and MCF7 cells, knocking down *KLLN* expression resulted in decreased Ser15-phosphorylation of p53 after doxorubicin treatment at all timepoints (1, 2, 4, and 8 h) as compared to control-transfected cells (Fig. 4b). Total p53 expression at these timepoints after treatment did not show any significant difference between the control-transfected and siKLLN-transfected cells (Fig. 4b). Since MDA-MB-231 cells have a mutant p53 with stable p53 expression, we assessed the effect of *KLLN* knockdown in these cells and found that silencing *KLLN* expression did not affect p53 phosphorylation after treatment. Further, MDA-MB-231 cells did not show a large increase in p53 phosphorylation or a change in total p53 expression in response to DNA damage (Fig. 4c). We also assessed the effect of knocking down *KLLN* expression on another DDR protein, CHK2. In MCF10A and MDA-MB-231 cells, silencing *KLLN* expression after treatment did not have any effect on the Thr68-phosphorylation of CHK2 or the total CHK2 expression at any of the timepoints tested (Fig S3). However, in MCF7 cells, we did observe a decrease in CHK2 phosphorylation (Thr68) in the siKLLN-transfected cells compared to the control-transfected cells at all timepoints tested, but no change in total CHK2 expression was observed (Fig S3). Therefore, KLLN regulation of DDR is more likely through the regulation of p53 activation rather than CHK2 activation.

### Loss of KLLN expression-associated decreased p53 phosphorylation not mediated by KLLN regulation of sensory DDR proteins

**Fig. 5:**
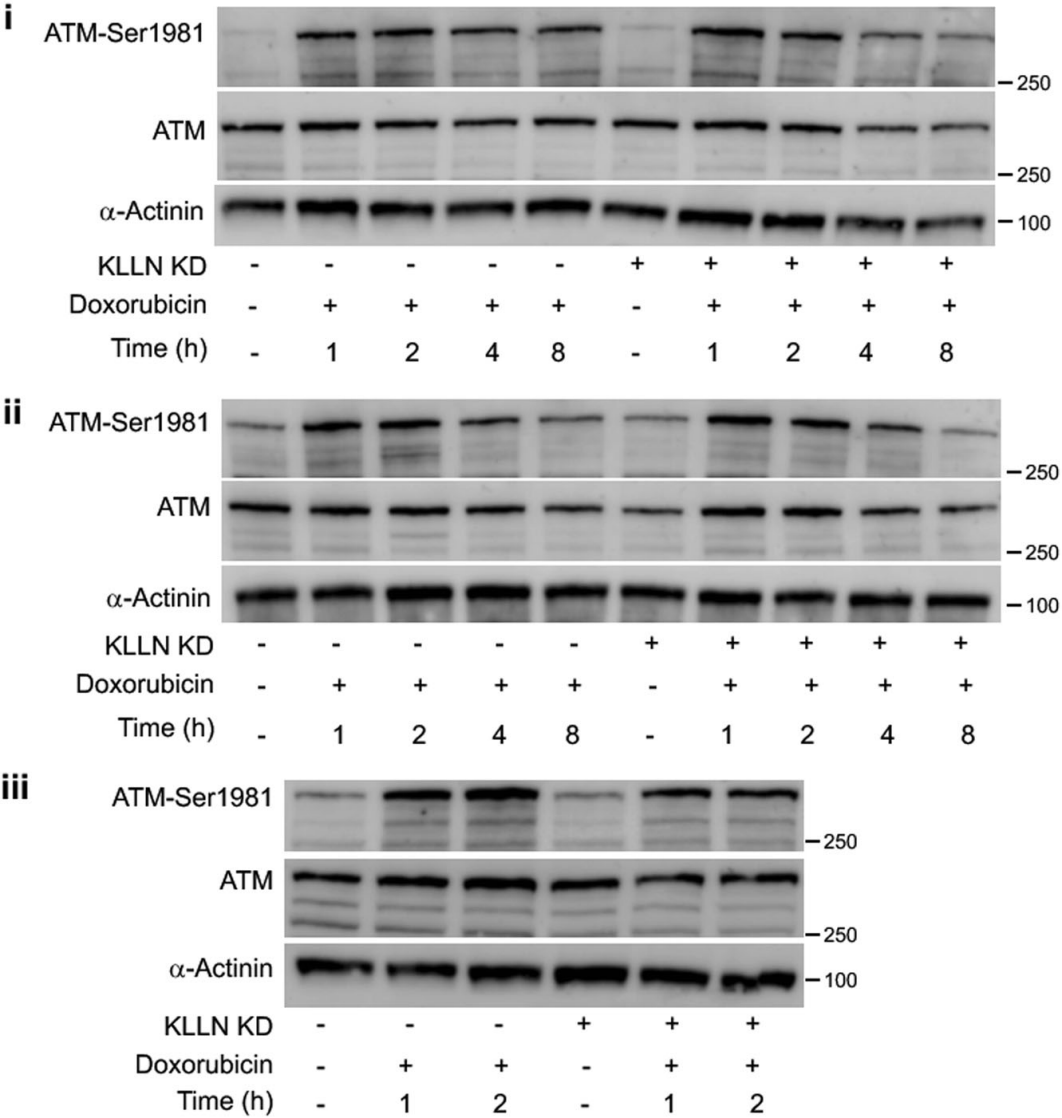
KLLN regulation of p53 phosphorylation is not through regulation of ATM phosphorylation. Immunoblotting for Ser1981-phosphorylation of ATM after doxorubicin-induced DNA damage showed that ATM phosphorylation was unaffected in MCF7 (**i**) and MDA-MB-231 (**ii**) cells after knockdown of *KLLN* expression. In MCF10A (**iii**) cells, knocking down *KLLN* expression resulted in a 30% decrease in phosphorylation of ATM after DNA damage

Since KLLN expression is associated with the regulation of p53 phosphorylation, we investigated KLLN regulation of upstream activators of p53 phosphorylation. In response to DNA damage, proteins like ataxia telangiectasia mutated (ATM) and ataxia telangiectasia and Rad3-related (ATR) sense DNA damage and are phosphorylated, thereby triggering a signaling cascade responsible for responding to the damage in an appropriate manner^24–28^. Phosphorylation of p53 is part of this signaling cascade; based on factors such as extent of damage and the phase of the cell cycle, the cell chooses various fates such as apoptosis and cell cycle arrest and subsequent DNA repair^21^. Therefore, we assessed the effect of knocking down *KLLN* expression on the phosphorylation status of both ATM and ATR after doxorubicin-induced DNA damage. We observed increased Ser1981-phosphorylation of ATM and no concomitant increase in Ser428-phosphorylation of ATR at early timepoints (1 and 2 h) after doxorubicin treatment in all cell lines (Fig. 5; Fig S4). In MCF7 and MDA-MB-231 cells, we also observed an increase in ATM phosphorylation at 4 h and 8 h after treatment (Fig. 5). Next, we assessed the effect of knocking down *KLLN* expression on the regulation of ATM phosphorylation. We observed that in MCF7 and MDA-MB-231 cells, silencing *KLLN* expression did not alter the Ser1981-phosphorylation of ATM after treatment at the timepoints tested (Fig. 5). There was no difference in the total expression of ATM either. In MCF10A cells, knocking down *KLLN* expression decreased the Ser1981-phosphorylation of ATM by 30% at 1 and 2 h after treatment (Fig. 5). Since ATR did not change in response to doxorubicin-induced damage, the change in expression of total and phosphorylated ATR after KLLN knockdown suggests that ATR does not contribute to KLLN’s role in p53 phosphorylation. Therefore, KLLN regulation of p53 phosphorylation is likely not through the regulation of sensory DDR proteins such as ATM and ATR.

### Loss of KLLN expression decreases phosphorylation of DBC1 at early timepoints after doxorubicin-induced DNA damage

**Fig. 6:**
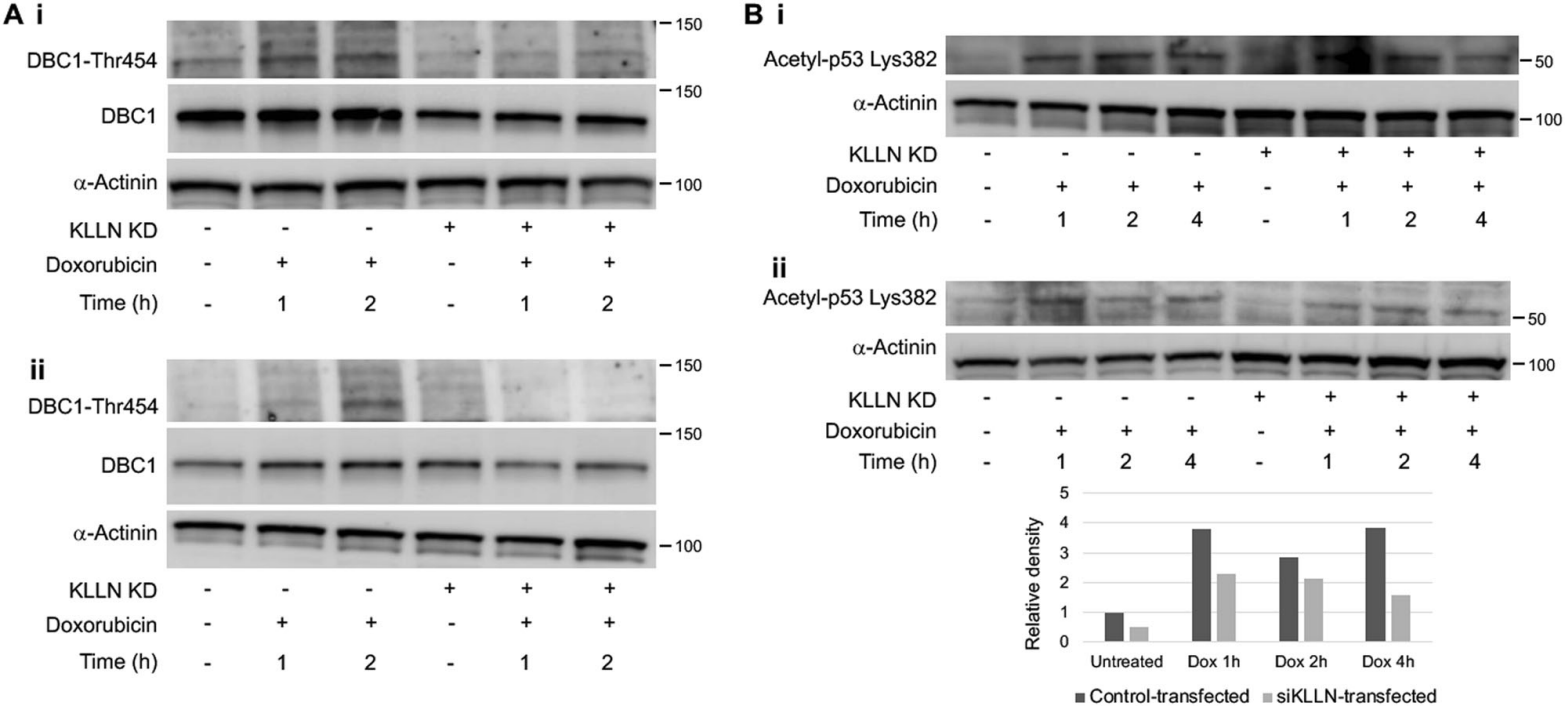
KLLN’s role in p53 acetylation after DNA damage is through the regulation of DBC1 phosphorylation. **a** Immunoblotting for Thr454-phosphorylation after doxorubicin-induced DNA damage showed that knocking down KLLN expression resulted in decreased DBC1 phosphorylation in MCF10A (i) and MCF7 (ii) cells. **b** Immunoblotting for Lys382-acetylation of p53 after doxorubicin-induced DNA damage showed decreased p53 acetylation after knockdown of KLLN expression in MCF7 (i) and MCF10A (ii) cells

SIRT1 is known to inhibit p53 activation by deacetylating p53 at Lys382^29–31^. Phosphorylation of deleted in breast cancer 1 (DBC1) at Thr454 in response to DNA damage results in the binding of DBC1 to SIRT1, thereby abrogating the inhibitory deacetylation of p53 by SIRT1^31,32^. We have shown previously that KLLN interacts with DBC1^10^; therefore, we assessed the effect of silencing *KLLN* on total DBC1 expression and its phosphorylation. We observed that knocking down *KLLN* expression after doxorubicin-induced DNA damage resulted in decreased Thr454-phosphorylation of DBC1 as well as total DBC1 expression in all three cell lines studied (Fig. 6a, Fig S5). We confirmed the effect of lack of phosphorylated DBC1 on p53 acetylation by assessing acetyl-p53 (K382) expression. We observed that knocking down *KLLN* expression followed by doxorubicin-induced DNA damage resulted in an expected decrease in Lys382-acetylation of p53 in MCF10A and MCF7 cells (Fig. 6b). Interestingly, in MDA-MB-231 cells (*TP53* mutated), we did not observe a decrease in p53 acetylation (Fig S5). This suggests that in cells with normal p53 function, KLLN likely regulates p53 activation in a two-pronged manner by aiding in damage-induced phosphorylation and maintaining the p53 acetylation in response to damage.

## Discussion

KLLN (or killin) was originally discovered as a target of p53 involved in S-phase cell cycle regulation and described to be necessary and sufficient for p53-mediated apoptosis^7^. These studies based their conclusions on lack of proliferation after overexpression of KLLN, and decreased cleavage of caspase-3 and PARP without a DNA damage background^7^. When these studies used a DNA damaging agent such as 5-Fluorouracil, cleavage of caspase 8 was assessed as their marker for apoptosis and showed decreased expression^7^. Cleaved caspase 8 is commonly activated by the extrinsic apoptotic pathway and not due to DNA damage. We, therefore, sought to address the role of KLLN in the intrinsic apoptosis pathway using doxorubicin as a DNA damaging agent. After confirming that increased *KLLN* expression in response to DNA damage could be abrogated by RNAi-mediated *KLLN* knockdown, we show here that lack of *KLLN* expression resulted in improper response to DNA damage evidenced by decreased frequency of γ-H2AX foci and γ-H2AX expression. Consequently, DNA damage-induced apoptosis is also decreased in our cells lacking KLLN expression as demonstrated by the decreased frequency of apoptotic nuclei and increased proliferation, and also by the decreased expression of definitive markers of apoptosis such as cleavage of caspase-3 and PARP.

The existing literature suggests a role for KLLN in S-phase cell cycle regulation, whereby these conclusions were based on the observation that the S-phase DNA content was unaffected after induction of KLLN expression and subsequent growth arrest^7^. The researchers had observed substantial apoptosis in this setting^7^. Similarly, G2 checkpoint dysfunction has also been observed in cells with naturally occurring germline variants of *KLLN*^5^. In our current studies, we observed that doxorubicin-induced DNA damage increased the cells in S and G2/M phases while knocking down *KLLN* did not change this frequency. These observations together with the results from Cho et al.^7^ suggest that KLLN most likely does not have a role in cell cycle regulation after DNA damage. In most cells, DDR in the form of cell cycle regulation is followed by DNA repair in an effort to save the cell. We had observed that knocking down *KLLN* expression in the setting of DNA damage results in increased genomic instability as observed by the increased frequency of micronuclei. This increased genomic instability is suggestive of the lack of repair after DNA damage in these cells and therefore, the cells are most likely lacking of upstream cell cycle regulation.

Our observations that KLLN was able to regulate apoptosis in the presence (MCF7 and MCF10A) and absence (MDA-MB-231) of wildtype p53 suggest that KLLN’s role in damage-induced apoptosis is likely p53-independent. Even though MDA-MB-231 has a mutant p53 status, this cell line expresses a p53 protein, which is known to contribute to survival signals and malignant progression^33,34^. Therefore, a cell line with a null p53 would be required to establish beyond a doubt of p53’s independence of KLLN’s role in DDR. Previous studies have suggested that KLLN is under the regulatory control of p53 since KLLN is a late by-product of the induction of p53 expression^7^. Subsequently, it has been shown that KLLN is capable of binding to the p53 promoter and regulating p53 expression^6,9^. Although our earlier results suggested a possible p53-independent role for KLLN in apoptosis regulation, the known regulatory loop between p53 and KLLN, and the critical role had by p53 in DNA damage-induced apoptosis led us to hypothesize that in cells with a wildtype p53, KLLN’s role in apoptosis could be through the regulation of p53. Hence, we investigated KLLN’s role in the regulation of p53 activation following DNA damage in cells with wildtype p53 and found that KLLN indeed does have a role. Acetylation and phosphorylation of specific p53 residues are known to stabilize the protein and prevent proteasomal degradation^35–37^. Previous mass spectrometry results of potential KLLN interactors revealed a disproportionate number of proteins involved in proteasomal degradation. Since both total p53 and total DBC1 showed some differences in expression between KLLN knockdown and control-transfected samples, a possible mechanism for KLLN regulation of these proteins could be through proteasomal degradation. Interestingly, KLLN expression did not show any association with the activation of some of the other common DDR proteins such as ATM, ATR, or CHK2.

**Fig. 7:**
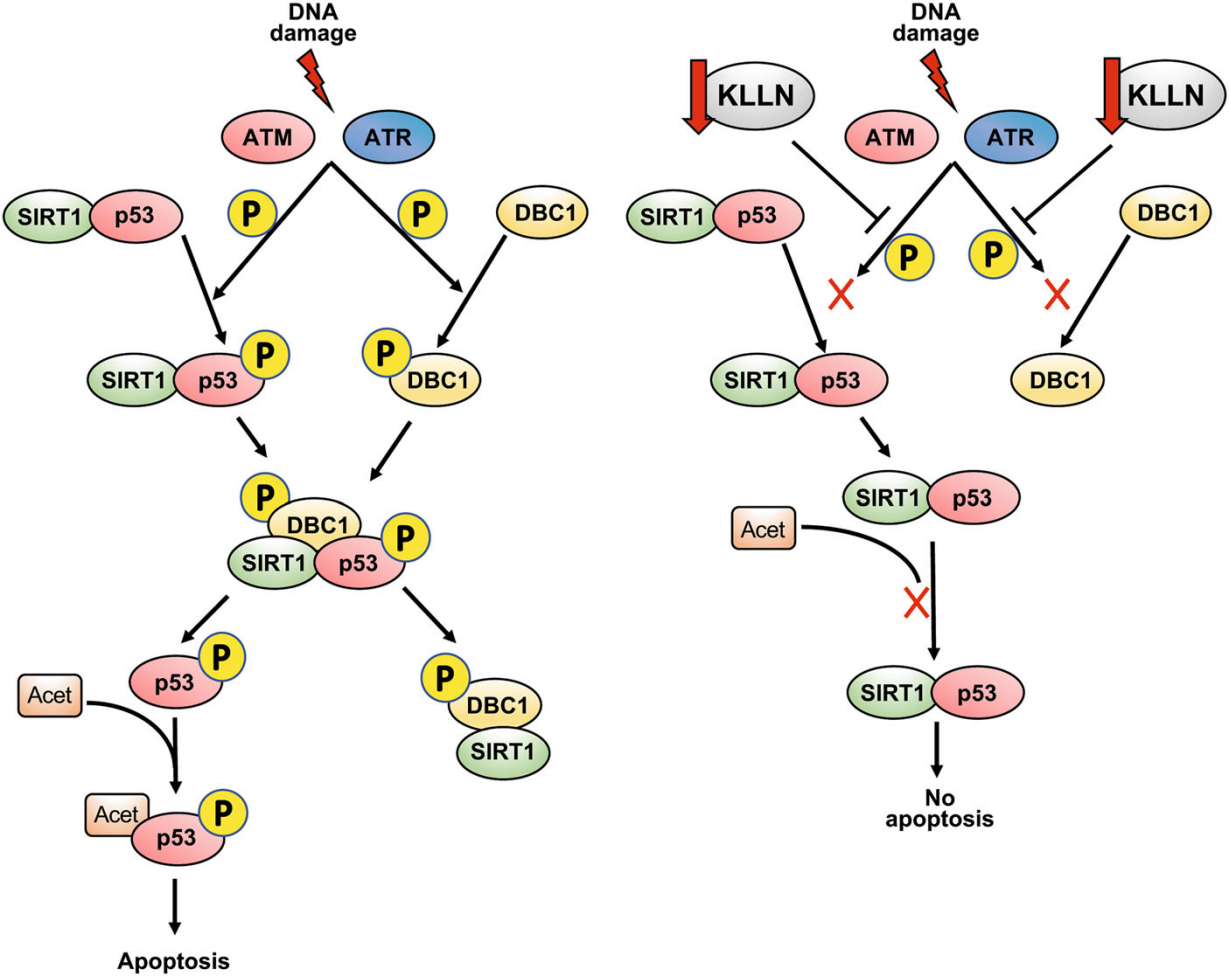
Schematic for KLLN regulation of p53 activation after DNA damage (modified from Zannini et al.,^32^). Knocking down KLLN results in the abrogation of p53 activation after DNA damage by decreasing both the phosphorylation and acetylation of p53

DBC1 is a nuclear protein that in response to DNA damage is phosphorylated at Thr454 by ATM and ATR, and this phosphorylation competitively inhibits SIRT1, a NAD^+^-dependent deacetylase^32^. Diminished expression of DBC1 results in increased SIRT1 activity and consequently increased deacetylation of p53 and abrogation of apoptosis^32^. Our previous results have shown that DBC1 interacts with KLLN and the KLLN–DBC1 interaction is possibly related to regulation of H3K9 trimethylation^10^. Our current assessment revealed that KLLN expression is associated with the regulation of DBC1 phosphorylation (Thr454) in response to DNA damage and thereby regulates p53 acetylation on Lys382. Therefore, our results suggest that KLLN may have a two-pronged role in regulation of p53 activation in response to DNA damage (Fig. 7). A potential mechanism for regulation of p53 activity might be through KLLN interaction with TRIM25, an E3 ligase, that is known to increase p53 expression but inhibit p53 activity^38^. Therefore, TRIM25 through modulating p53 signal is capable of enhancing cell survival, at least in prostate cancer cells^39^. We postulate that KLLN may be able to sequester TRIM25 and abrogate its inhibitory effect on p53.

A technical limitation of our study is the lack of a trustworthy KLLN antibody. This observation has also been previously noted^40^. Therefore, we were unable to confirm the protein expression of KLLN in our studies. We have tested every available commercial antibody and none passed our intense quality control protocol since in most cases the antibodies were unable to detect FLAG or GFP-tagged KLLN protein nor were these antibodies able to detect expression differences among different tissue types. Some of these antibodies showed reactivity to mouse and rat samples, even though it is known that a mouse or rat homolog of the KLLN protein does not exist. The inability to produce a KLLN-specific antibody is directly related to the difficulty associated with its purification. KLLN expressed in bacteria are known to adopt a conformation that has very low affinity for purification columns, making the purification of the native protein close to an impossibility^7,41^. Also, bioinformatic analysis of the protein suggests that KLLN has a large number of trypsin cleavage sites that results in the fragmentation of the protein, thereby making the purification of KLLN especially challenging. In lieu of the KLLN protein data, we used the qRT-PCR data to report *KLLN* gene expression after DNA damage and RNAi-mediated silencing as a surrogate.

In conclusion, our data establish a role for KLLN in DNA damage-induced apoptosis through the regulation of p53 phosphorylation and acetylation. We were also able to demonstrate that KLLN’s role in apoptosis regulation may be independent of p53. These data also close a regulatory loop between p53 and KLLN where both proteins are capable of regulating the expression and activation of each other in stress conditions. From a translational point of view, since KLLN can function in the absence of p53, inhibiting KLLN function could induce synthetic lethality in the context of both germline and somatic *TP53* mutations.

## Materials and methods

### Cell culture

MCF7 and MDA-MB-231 breast cancer cells were cultured in DMEM media supplemented with 10% FBS (Life Technologies, Carlsbad, CA). MCF10A breast epithelial cells were cultured in MEBM media (Lonza, Walkersville, MD) supplemented with components of the MGEM bulletkit (Lonza) and cholera toxin (100 ng/ml) (Sigma Aldrich, St. Louis, MO). Cell lines were cultured at 37 °C and 5% CO_2_ and passaged using Trypsin-EDTA. All cell lines were purchased from ATCC (Manassas, VA) after 2010 and authenticity was documented by standard STRS analysis per ATCC routine. All cell lines were used during passage 3 to 20 and routinely tested for mycoplasma.

### Doxorubicin treatment

Doxorubicin hydrochloride (Sigma Aldrich) was used to induce DNA damage in our breast normal and cancer cells. A 2 µM dose of doxorubicin was considered as the appropriate dose for our studies. For knockdown experiments, doxorubicin was added 48 h after transfection with siRNA.

### RNAi-mediated silencing of KLLN expression

For transfection of siRNA, cells were seeded at 50–60% confluence in appropriate dishes and allowed to attach overnight. For KLLN knockdown, cells were transfected with KLLN siRNA smartpool (GE Dharmacon, Lafayette, CO) using Lipofectamine RNAimax (Thermo Fisher Scientific, Waltham, MA) according to the manufacturer’s instructions. A scrambled siRNA pool (GE Dharmacon) was used as a control. On the basis of previous studies, 48–72 h after transfection was considered as peak knockdown and QRT-PCR was used to confirm knockdown of KLLN expression.

### RNA collection, reverse transcription, and quantitative PCR

RNA was collected using the Nucleospin RNA plus kit (Takara Bio USA, Mountain View, CA) and RNA concentration quantified using a Nanodrop spectrophotometer (Thermo Fisher Scientific). Reverse transcription was done using Primescript RT reagent kit (Takara Bio USA) following the manufacturers protocol. cDNA was quantified using SYBR Green PCR Master Mix (Life Technologies) on the 7500 Real Time PCR System (Applied Biosystems, Foster City, CA) using primers specific for KLLN and GAPDH. Data were analyzed using the standard 2^-ΔΔCT^ method.

### Immunofluorescence

For immunofluorescence, cells were grown on coverslips in a 6-well plate and transfected with *KLLN* siRNA. Forty-eight hours after transfection, cells were treated with the appropriate dose of doxorubicin or left untreated. After specific intervals of times with treatment, cells on the coverslips were washed with 1×PBS and fixed with 3.7% formaldehyde for 15 min at room temperature. After washing with 1×PBS, cells were permeabilized in 0.3% Triton-X in 1×PBS for 5 min at room temperature. After another washing step, cells were blocked in 10% normal goat serum for 1 h at room temperature before being incubated overnight at 4 °C with primary antibodies diluted in blocking solution: phospho-H2AX (ser139) at 1:1000 (Millipore, Burlington, MA; cat# 05–636). Coverslips were again washed with 1×PBS and incubated for 1 h with secondary antibodies in 0.3% triton PBS: Alexa Fluor 488 goat anti-mouse IgG (cat# R37120) at 1:1000 (Thermo Fisher Scientific). After washing, coverslips were mounted onto slides with DAPI-containing mounting media (Vector Laboratories, Burlingame, CA). Slides were blinded and images were analyzed using upright confocal microscopy (Leica Microsystems, Buffalo Grove, IL) and Leica Confocal Software for image analysis. The number of cells with and without >20 foci were enumerated. A ratio between the two was calculated and plotted on a graph.

### TUNEL assay

For TUNEL assay, cells were also grown on coverslips, and transfections and treatments were done as described for immunofluorescence. After the appropriate intervals of times with treatment, cells were fixed and permeabilized as described for immunofluorescence. TUNEL assay was done using the in-situ cell death detection kit, TMR red (Sigma Aldrich) according to manufacturer’s instructions. After staining, coverslips were mounted onto slides with DAPI-containing mounting media (Vector Laboratories). Slides were blinded and images were analyzed using upright confocal microscopy (Leica Microsystems) and Leica Confocal Software for image analysis. The number of apoptotic nuclei were enumerated and plotted on a graph.

### Cell viability assay

For cell viability assay, cells were grown in 6-well plates, and transfections and treatments were done as described for immunofluorescence. After the appropriate intervals of times with treatment, cells were harvested and counted on a Countess^TM^ automated cell counter (Thermo Fisher Scientific) according to manufacturer’s instructions. The number of viable cells were enumerated and plotted on a graph.

### Western blotting

Cells harvested after transfection and doxorubicin treatment were lysed using Mammalian Protein Extraction Reagent (Pierce, Rockford, IL) supplemented with protease and phosphatase inhibitors (Sigma Aldrich). Protein concentrations were quantified using the BCA Protein Assay (Pierce) according to manufacturer’s instructions. Overall, 20–40 μg of protein mixed with a 2× loading dye containing β-mercaptoethanol was heated at 100 °C for 10 min and run on a Criterion^TM^ 4–15% gradient gel (Bio-Rad, Hercules, CA). For high molecular weight (HMW) proteins, the samples (40 μg) were run on Criterion^TM^ TGX^TM^ 7% gel. Protein was transferred to a nitrocellulose membrane using Trans-Blot Turbo (Bio-Rad) according to manufacturer’s instructions and membranes blocked for 30 min–1 h in 3% BSA. HMW proteins were transferred to activated PVDF (using 100% methanol) membranes using Trans-Blot Turbo. For these HMW proteins, ethanol was not added to the transfer buffer. After transfer, membranes were dipped in 100% methanol for 10 s, dried for a 3–5 min and then washed with 20% methanol for 5 min. Membranes were then washed three times with 1×TBST and blocked in 3% BSA for 1 h. Membranes were incubated overnight with primary antibody at 4 °C. Blots were washed the next day using 1×TBST (three times, 5 mins each) and incubated with secondary antibody for 1 h at room temperature. Superscript West Pico chemiluminescent substrate (Thermo Fisher Scientific) or Clarity western ECL substrate (Bio-Rad) was used for chemiluminescent detection and images were captured using Amersham imager 600 (GE Healthcare Sciences, Marlborough, MA). Densitometric analysis was done using the NIH software ImageJ.

Primary antibodies commonly used in this study were for p53 (1:1000) (Santa Cruz Biotechnology, Santa Cruz, CA, cat# sc126), phospho-p53 (Ser15) (1:1000) (Cell signaling, cat#9284), phospho-H2AX (Ser139) (1:1000) (Millipore, cat# 05–636), cleaved caspase-3 (1:1000) (Cell signaling, cat# 9664), DBC1 (1:10,000) (Bethyl Labs, cat# A300-432A), phospho-DBC1 (1:750) (Thr454) (Cell signaling, cat# 4880), PARP (1:1000) (Cell signaling, cat# 9542), CHK2 (1:1000) (Cell signaling, cat# 2662), phospho-CHK2 (Thr68) (1:1000) (Cell signaling, cat# 2661), ATM (1:1000) (Cell signaling, cat# 2873), phospho-ATM (1:1000) (Cell signaling, cat#13050), ATR (1:1000) (Cell signaling, cat#2790), phospho-ATR (Ser428) (1:1000) (Cell signaling, cat# 2853). Alpha actinin (1:1000) (Cell signaling, cat# 3134), and alpha tubulin (1:10,000) (Sigma, cat# T6074) were used as loading controls. Secondary antibodies used were anti-mouse HRP and anti-rabbit HRP (1:2500) (Promega, cat#s W4021, W4011).

### Statistical analysis

All experiments in this study were performed in duplicate or in triplicate. All data collected from these in vitro studies were analyzed by two-tailed Students *t*-tests. Significance of results was determined based on calculated *p*-values and a *p* value < 0.05 was considered statistically significant. All data were represented with error bars signifying standard error of mean for population mean and standard deviation for sample mean.

## Electronic supplementary material

Supplemental Data

Author contribution form
