# Supplementary material for: KLLN-mediated DNA damage-induced apoptosis is associated with regulation of p53 phosphorylation and acetylation in breast cancer cells
Source: Cell Death Discov. 2018 Sep 11;4:92. doi: 10.1038/s41420-018-0094-x (PMC6134104; doi:10.1038/s41420-018-0094-x)
Supplement: Supplementary file 1 — Supplemental Data [file 41420_2018_94_MOESM1_ESM.docx]

**SUPPLEMENTAL INFORMATION**

**TITLE: KLLN-mediated DNA damage-induced apoptosis is associated with regulation of p53 phosphorylation and acetylation in breast cancer cells**

**AUTHORS:** Madhav Sankunny^1,2^, Charis Eng^1,2,3,4,5,*^

**Supplementary Figures and Legends**


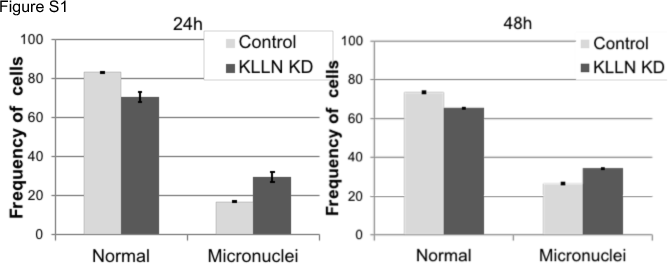


**Supplementary Figure S1.** RNAi-mediated silencing of *KLLN* expression after DNA damage (24 and 48h) increases genomic instability as measured by micronuclei frequency in MDA-MB-231 cells. Values represent mean ± SD.


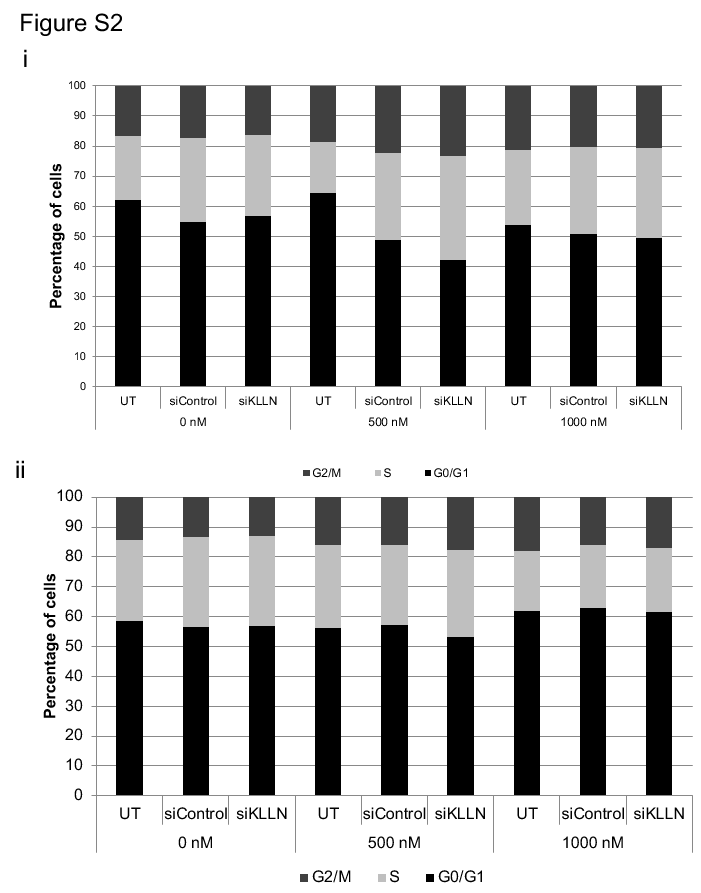


**Supplementary Figure S2.** Knock down of *KLLN* expression after DNA damage does not affect cell cycle regulation. Cell cycle analysis using propidium iodide staining and flow cytometry in MCF7 (i) and MDA-MB-231 (ii) cells showed that doxorubicin-induced DNA damage increased S and G2/M phase proportion but was unaffected by the knock down of *KLLN* expression. Values represent mean.


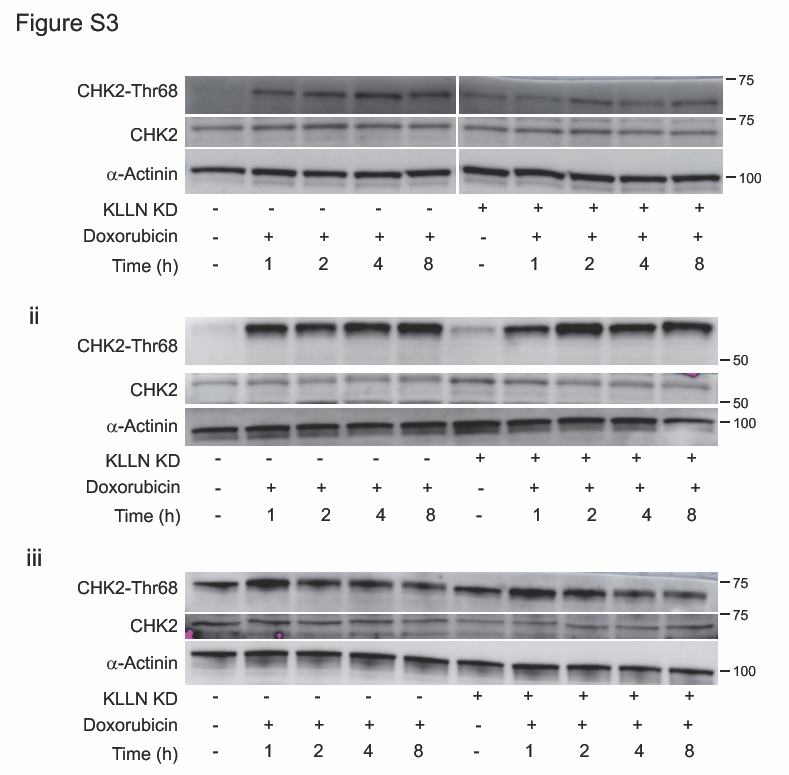


**Supplementary Figure S3.** Immunoblotting for Thr68-phosphorylation of CHK2 showed that knock down of *KLLN* expression does not affect activation of CHK2 early in response to DNA damage-induced by doxorubicin in MCF7 (i), MCF10A (ii) and MDA-MB-231 (iii) cells.


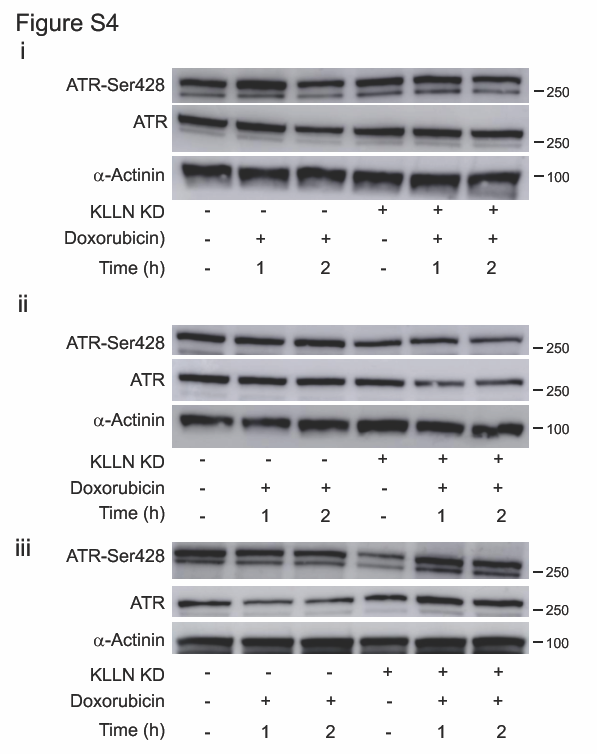


**Supplementary Figure S4.** Immunoblotting for Ser428-phosphorylation of ATR showed that ATR phosphorylation was unaffected by DNA damage-induced by doxorubicin in MCF7 (i), MCF10A (ii) and MDA-MB-231 (iii) cells.


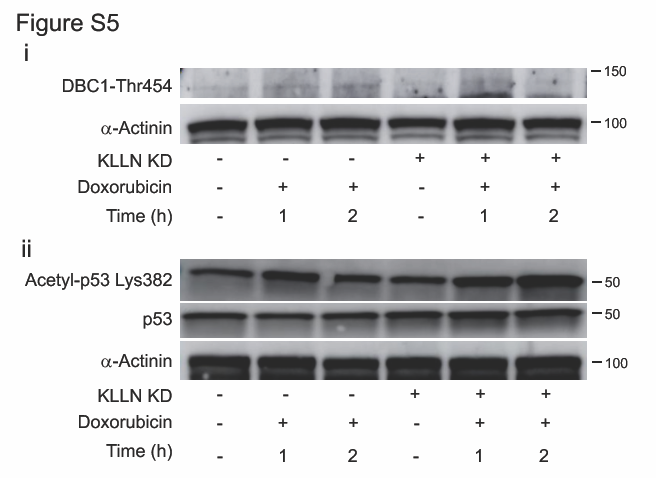


**Supplementary Figure S5.** Immunoblotting for (i) Thr454-phosphorylation of DBC1 and (ii) Lys382-acetylation of p53 showed that knock down of *KLLN* expression decreased DBC1 phosphorylation but increased p53 acetylation after DNA damage-induced by doxorubicin in MDA-MB-231 cells.


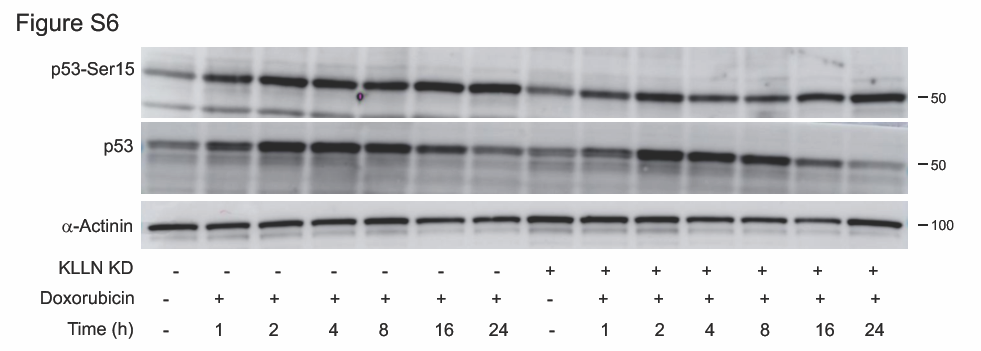


**Supplementary Figure S6.** Immunoblot of Ser15-phosphorylation of p53 for MCF7 cells before splicing for Figure 4B (i).
