# Supplementary material for: KLLN-mediated DNA damage-induced apoptosis is associated with regulation of p53 phosphorylation and acetylation in breast cancer cells
Source: Cell Death Discov. 2018 Sep 11;4:92. doi: 10.1038/s41420-018-0094-x (PMC6134104; doi:10.1038/s41420-018-0094-x)
Supplement: Supplementary file 2 — Author contribution form [file 41420_2018_94_MOESM2_ESM.pdf]

DECLARATION OF CONTRIBUTIONS TO ARTICLE

ADMC

Manuscript Number:	Journal Name:	
	Cell Death & Differentiation	(the 'Journal')

Proposed Title of the Contribution:

KLLN-mediated DNA damage-induced apoptosis is associated with regulation of p53 phosphorylation and acetylation in breast cancer cells (the 'Contribution)

Author(s):

Madhav Sankunny, Charis Eng (the 'Authors')

For all *CDD* articles, each person named as an author in the published version must be able to show he or she has contributed substantially to the article.

Authorship credit should be based on 1) substantial contributions to conception and design, acquisition of data, or analysis and interpretation of data; 2) drafting the article or revising it critically for important intellectual content; and 3) final approval of the version to be published. Authors should meet conditions 1, 2 and 3.

Any person who cannot be shown to have made a substantial contribution to the article cannot be listed as an author in the final version. The name of any person who is deemed to have made a minor contribution can, however, appear in the Acknowledgments section of the article.

Please complete the table below to indicate the contributions of all named authors to the manuscript.

Author Full Name:	Specification of Contribution to the Manuscript:
Madhav Sankunny	1, 2 and 3
Charis Eng	1, 2 and 3

Please complete the table below to indicate the contributions of all named authors to the figures.

Figure 1:

MS - design, acquisition, analysis and interpretation of data
CE - design, analysis and interpretation of data

Figure 2:

MS - design, acquisition, analysis and interpretation of data
CE - design, analysis and interpretation of data

Figure 3:

MS - design, acquisition, analysis and interpretation of data
CE - design, analysis and interpretation of data

Figure 4:

MS - design, acquisition, analysis and interpretation of data
CE - design, analysis and interpretation of data

Figure 5:

MS - design, acquisition, analysis and interpretation of data
CE - design, analysis and interpretation of data

Figure 6:

MS - design, acquisition, analysis and interpretation of data
CE - design, analysis and interpretation of data

Signed for and on behalf of the Author(s):	Print Name:	Date:
	Charis Eng	May 4, 2018
